# Mutans streptococci genetic strains in children with severe early childhood caries: follow-up study at one-year post-dental rehabilitation therapy

**DOI:** 10.3402/jom.v4i0.19530

**Published:** 2012-12-14

**Authors:** Elizabeth A. Palmer, Alex Vo, Shelby B. Hiles, Patricia Peirano, Samyia Chaudhry, Amy Trevor, Iraj Kasimi, Jill Pollard, Christopher Kyles, Michael Leo, Beth Wilmot, John Engle, John Peterson, Tom Maier, Curtis A. Machida

**Affiliations:** 1 Department of Pediatric Dentistry, Oregon Health & Science University (OHSU) School of Dentistry, Portland, OR; 2 Academic DMD Program, OHSU School of Dentistry, Portland, OR; 3 Integrative Biosciences, OHSU School of Dentistry, Portland, OR; 4 OHSU School of Nursing, Portland, OR; 5 Oregon Clinical and Translational Research Institute, OHSU, Portland, OR; 6 Division of Bioinformatics and Computational Biology, Department of Medical Informatics and Clinical Epidemiology, OHSU School of Medicine, Portland OR; 7 Oral Pathology and Radiology, OHSU School of Dentistry, Portland, OR

**Keywords:** mutans streptococci, distribution of genotypic MS strains, *Streptococcus mutans*, oral streptococci, severe early childhood caries, full-mouth dental rehabilitation therapy

## Abstract

**Background:**

Genotypic strains of cariogenic mutans streptococci (MS) may vary in important virulence properties. In previous published studies, we identified 39 MS strains from pediatric patients undergoing full-mouth dental rehabilitation, including the removal and/or repair of carious lesions and application of antimicrobial rinse and fluoride varnish.

**Objectives:**

The objectives of this current 1-year follow-up study are to assess the variability of MS strains that occur at 1-year post-rehabilitation and characterize the xylitol-resistance properties of MS strains that predominate.

**Methods:**

Plaque from five children with severe early childhood caries was collected 1-year post-rehabilitation. MS isolates were subjected to arbitrarily primed-polymerase chain reaction (AP-PCR) for identification of genetic strains and *in vitro* xylitol-inhibition experiments. To more precisely define strain distributions within each patient, we isolated large numbers of isolates per patient.

**Results:**

MS strains diminished from several strains pre-rehabilitation, to one dominant strain at 1-year post-rehabilitation, with several new emergent strains. The majority of the clinical MS strains, as well as the *Streptococcus mutans* laboratory strains ATCC 25175 and 35668, were predicted to undergo 50% inhibition with 2.48–5.58% xylitol, with some clinical MS strains being significantly more resistant *in vitro*.

**Conclusions:**

Our follow-up study using patients from the original cohort demonstrates that specific MS strains are dominant at 1-year post-dental rehabilitation. Most of the clinical MS strains are similar in xylitol resistance to the attenuated *S. mutans* ATCC control strains, with some strains being more resistant to xylitol *in vitro*.

Dental caries represents one of the most common chronic diseases affecting young children (1), and is a multifactorial disease involving complex interactions of genetic, dietary, environmental and behavioral risk factors (2). Shifts in the pH and physiological condition of the oral cavity may lead to changes in the microbial composition of the oral biofilm (2). The acidogenic MS group, including *S. mutans* and *Streptococcus sobrinus*, are among the cariogenic microorganisms that reside in the oral cavity, and represent the most predominant bacteria associated with dental caries. The oral cavity harbors distinct genotypes of *S. mutans* in saliva and dental plaque, with at least 52 known genotypes (3, 4). Some MS genotypes may have preferential abilities to colonize and predominate in specific oral environments (3–5). Individuals with high caries versus low caries prevalence may retain MS strains exhibiting distinct differences in virulence and caries-promoting activities (1). The coexistence and concurrent virulence of multiple MS genotypes in caries-active individuals may serve as important determinants for increased caries incidence, as well as treatment success or failure (5).

Caries formation starts with the accumulation of salivary proteins and adhesive glucans on the enamel surface to form the oral biofilm, thereby allowing mutans streptococci (MS) adherence to the tooth surface (1, 6, 7). Lactic acid production during carbohydrate metabolism by MS causes demineralization of the local enamel and may eventually lead to decalcification, dissolved tooth structure and potential tooth loss. Accumulation of MS can alter the pH of the oral biofilm, which can subsequently select for increased numbers of acidogenic microorganisms.

Xylitol is a five-carbon sugar alcohol, which is capable of reducing plaque formation, inhibiting enamel demineralization by reducing acid generation, and suppressing growth of plaque bacteria, including MS. It has been proposed that the inhibitory effects of xylitol on MS may be due to the intracellular accumulation of xylitol 5-phosphate and subsequent competition with bacterial glycolytic enzymes [e.g. phosphofructokinase], arrest of glycolysis, and impaired growth (8). The inhibitory effect of xylitol on enamel demineralization may potentially have an additive effect with fluoride (9). In some cases, long-term xylitol consumption has led to the selection of xylitol-resistant MS strains (10), with distinct agglutination, aggregation and adherence properties (11–13). For these xylitol-resistant MS strains, bacteria may be more readily shed into saliva, resulting in the reduction of MS numbers in plaque and potentially hampering transmission and/or colonization between individuals (14–16).

Xylitol-containing products are frequently used in caries prevention practices. As the use of these products continues to gain popularity, questions emerge concerning the potential adaptation of plaque microorganisms, including MS, to xylitol and the possible selection of xylitol-resistant MS strains with increased cariogenic potential. Studies conducted in young children, as opposed to studies conducted with adults, indicate that xylitol (40% solution) may not significantly suppress *Streptococcus mutans* counts or plaque accumulation (17). Thus, caries-active children may potentially possess distinct MS genetic strains with differential xylitol resistance properties, with some strains exhibiting increased cariogenic potential. One objective of this 1-year follow-up study was to provide insight into the use of xylitol treatment as an effective maintenance practice for caries preventive therapy in pediatric dental patients.

In this 1-year follow-up study, we examined the profiles of MS genotypic strains from remaining members of the pediatric patient cohort that had been diagnosed with severe early childhood caries [S-ECC]. In the original study, isolates were collected both prior to and following full-mouth dental rehabilitation, which included the removal and/or repair of carious lesions and application of antimicrobial rinse and fluoride varnish. We now describe the MS genetic strains that are dominant at 1-year post-dental rehabilitation and the emergence of six new previously undetected minor MS strains. We also now characterize the xylitol resistance properties of select dominant and minor strains.

## Methods

### Patient selection and treatment

As described in our previous report (18), participants for this research study were selected from patients attending the OHSU Pediatric Dentistry clinic. Approval for the use of human participants was obtained from the OHSU Institutional Review Board (IRB), and written informed consent was obtained from parents or guardians of the children participating in this study. The inclusion parameters for recruitment were young children in good general health, while children who had been subjected to antibiotic treatment, topical fluoride application, and/or antiseptic mouth rinses within the previous 3 months, or undergoing orthodontic therapies, were excluded. We selected participants who underwent full-mouth dental rehabilitation therapy, conducted under general anesthesia at Doernbecher Children Hospital [located within OHSU], because this allowed for full-mouth caries restorative therapy to be completed during a single patient visit. These individuals were within 3–5 years of age.

The patient demographics and decayed, missing and filled teeth (dmft) and decayed, missing and filled surfaces (dmfs) scores have been reported previously (18). Observations from the 1-year recall exam are also described in Table 1. Dental rehabilitation therapy included application of 0.12% chlorhexidine gluconate to the gingiva and dentition using a sterile gauze to prepare the surgical area prior to beginning the procedure, followed by amalgam (Valiant^®^ PH.D^®^), composite (Pulpdent^®^ Etch-Rite 38% Phosphoric Acid Etching Gel, Optibond™, Z100™ and Filtek™ Supreme), and stainless steel crown (3M ESPE, Unitek) restorations, formocresol (Patterson Dental) pulpotomies, extractions, sealants (Patterson Dental), dental prophylaxis (NUPRO^®^ prophylaxis paste [1.23% fluoride]), and sodium fluoride varnish (Cavity Shield^TM^) application with a brush.

**Table 1:** Demographics of patients (G, J, K, L and M) returning for one-year recall exam

		G	J	K	L	M
		
	Sex	Female	Male	Male	Male	Male
	Treatment age^1^	5 yr	5 yr	3 yr	3 yr	5 yr
	Teeth present^2^	A–T	A–T	A–T	A–T	A–T
Treatment day	dmft score^3^	11	13	18	13	12
	dmfs score^4^	25	38	61	48	41
						
	Teeth present^2^	A–K, M–T	3, A–C, H–J, 14, 19, K–M, 23–26, R–T, 30	A–T	A–T	A–C, H–T
	Condition of restorations	Satisfactory	Satisfactory	Satisfactory	Missing 1 restoration	Satisfactory
1-Year recall exam	No. of new carious lesions	1	2	1	2	1
	Lesion at margin of existing restoration?	Yes	No	No	Yes	No

1 Treatment age: Age of patient on day of full-mouth dental rehabilitation.

2 Teeth present: Teeth present on day of either full-mouth dental rehabilitation or at the 1- year recall exam. Letters denote primary teeth and numbers denote permanent teeth present following the Primary Universal Numbering System.

3 dmft: The sum of the primary teeth that are decayed (d), missing (m) or filled (f) due to dental caries.

4 dmfs: The sum of the primary tooth surfaces that are decayed (d), missing (m) or filled (f) due to dental caries.

Note: Table reproduced in part from Palmer et al. (18) with the kind permission of the American Academy of Pediatric Dentistry.

### Sampling procedure and processing of specimens

For this 1-year follow-up study, plaque samples were taken from each participant during the 1-year recall visit and compared to specimens collected at three previous time points: (1) prior to the initiation of dental rehabilitation therapy; (2) within the 2–4 weeks post-rehabilitation visit; and (3) at the 6-month recall visit. Plaque samples from each patient were individually collected by one of two clinicians, who received training on standardized procedures to achieve calibration for specimen collection. Two plaque samples were taken from each patient at each collection time point. Each plaque sample was obtained using a disposable sterile swab that was brushed along the buccal and lingual surfaces of the entire dentition.

### Control streptococci strains and selection of MS isolates

Control streptococci strains include *S. mutans* ATCC strains 25175 and 35668, *Streptococcus sobrinus* ATCC 33478, and non-MS oral streptococci strain *Streptococcus salivarius* ATCC 13419. As described in Fazilat et al. (19) and Palmer et al. (18), plaque specimens were plated on mitis salivarius agar (MSA; product number 229810, Difco, Becton, Dickinson and Company, Sparks, MD), supplemented with 1% sodium tellurite and the antibiotic bacitracin (0.2 U/ml), to isolate MS. Colonies were allowed to grow on MSA plates for 48–72 hours (37°C, 5% CO_2_), and then individual colonies were selected based on typical MS morphology (e.g. small, hard and shiny appearance) and propagated in brain heart infusion (BHI) broth for 24–48 hours at 37**°**C in 5% CO_2_. Cultures were then subjected to Gram stain analyses for verification of isolates as Gram-positive cocci.

### Genomic DNA isolation, conventional polymerase chain reaction (PCR) and arbitrarily primed PCR (AP-PCR)

As described in Palmer et al. (18), genomic DNA was extracted from overnight liquid cultures using the PureLink Genomic DNA Kit (Invitrogen) and *S. mutans* were independently identified using conventional PCR. Highly-specific primers for *S. mutans* and *S. sobrinus*, in addition to thermal cycling parameters, have been defined (20, 21), and are also described in our previous report (18). The amplification parameters for AP-PCR were similar to conventional PCR, with the exception of annealing at reduced temperatures (35**°**C for 30 seconds). PCR products were subjected to agarose (0.8%) gel electrophoresis, and ethidium bromide-stained fragments were visualized by UV transillumination.

### Xylitol-inhibition experiments and statistical curve-fitting analyses

For xylitol-inhibition experiments, MS genetic strains were grown overnight in BHI broth, prior to use in growth curve experiments measuring absorbance (*A*=600 nm) from 0 to 10 hours and then at 24 hours. Independent cultures were treated with varying xylitol concentrations (0–5%), with replicates (*n*=4) for each time point and xylitol concentration. Xylitol concentrations were selected based on concentrations used in similar *in vitro* xylitol inhibition assays (22) and may mimic in some cases the effective dose of xylitol in saliva released from oral xylitol-containing products. Curve fitting analysis using cubic and quadratic models (23–25) was conducted to determine the xylitol concentration (w/v) that resulted in 50% inhibition of growth, using the absorbance value of the 0% xylitol control at peak logarithmic phase (typically at 9–10 hours) for normalization at 100%.

## Results

### Description of study participants

Nine patients were originally enrolled in this study; seven (Patients G, H, I, J, K, L and M) were available for their initial 2–4 week recall visits and five (Patients G, J, K, L and M) were available for their 6-month and 1-year post-dental rehabilitation visits. In Table 1, for this current report, we now describe information collected from the five patients who completed the 1-year post-rehabilitation visits. The pediatric dentistry patients described in the pilot study were between the ages of 3 and 5 years old on the day of dental rehabilitation and were in good health (American Society of Anesthesiologists [ASA] physical status I. All patients, with the exception of Patient H, had a full complement of 20 primary teeth present. The patients were all diagnosed with severe-early childhood caries (S-ECC) with dmft and dmfs scores ranging from 11 to 18 and 25–61, respectively (18) (see also Table 1). The American Academy of Pediatric Dentistry AAPD) defines S-ECC in children ages 3–5 years old as: one or more cavitated, missing (due to caries) or smooth filled surfaces in primary maxillary anterior teeth, or dmfs score of >4 (age 3), >5 (age 4), or >6 (age 5) (26). All patients in our study underwent full-mouth dental rehabilitation therapy under general anesthesia.

### Identification of mutans streptococci strains

**Fig. 1:**
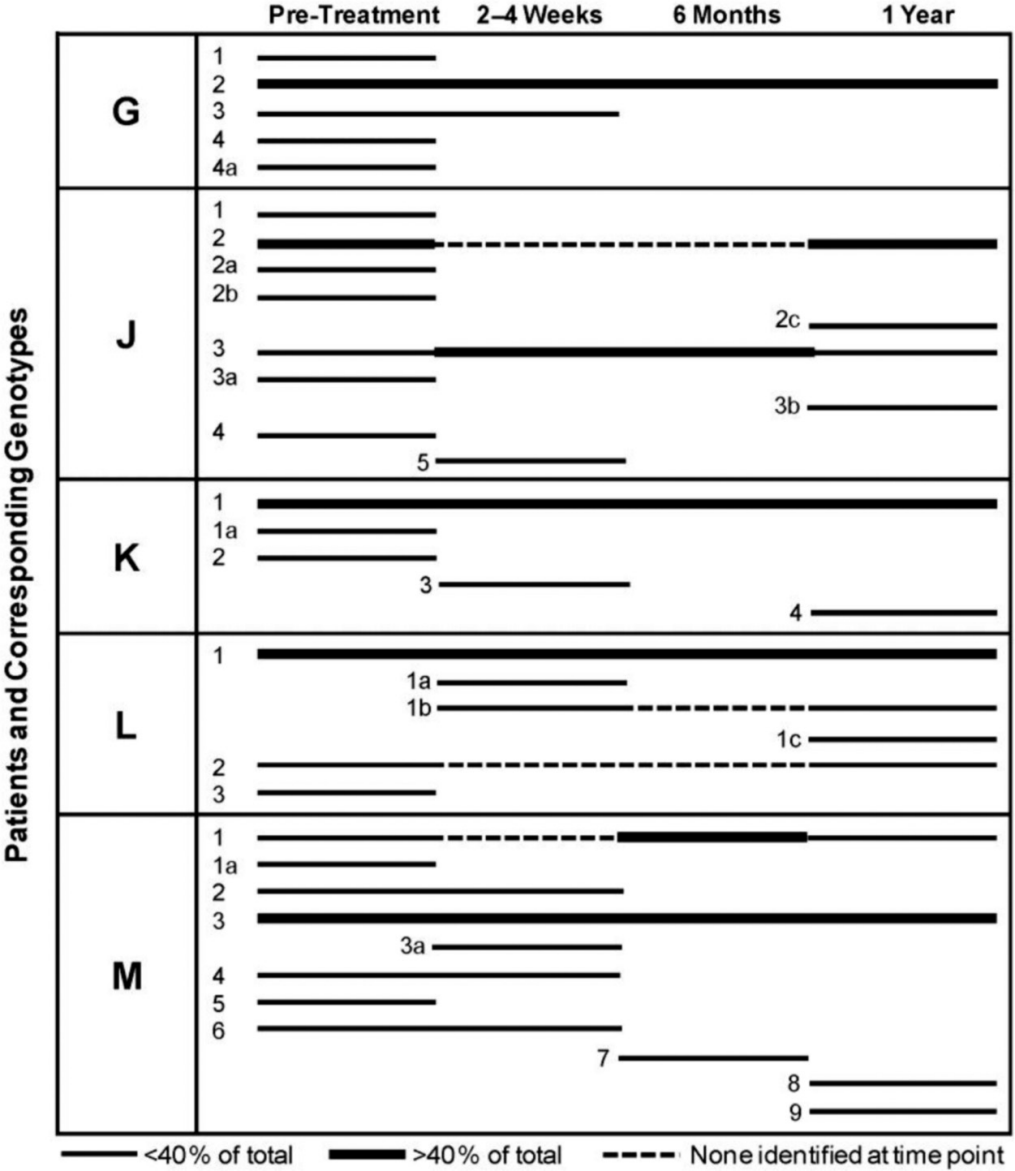
Genotypic strain diversity in pediatric dentistry patients at pre- and post-dental rehabilitation therapy (2–4 weeks, 6 months and 12 months). Each line represents distinct genotypes identified by AP-PCR. The dominant genotypes are marked in bold at each collection point (>40% of the isolates). Dotted lines indicate the periods when genotypes were detected at one time point but were not detected at subsequent time points. Isolates were genotyped numerically within each patient (G, J, K, L and M). Only data for Patients G, J, K, L and M, who completed all recall visits, including the 12-month post-therapy visit, are included in this analysis. Portions of this figure were previously displayed in Palmer et al. (18) and are reproduced with the kind permission of the American Academy of Pediatric Dentistry.

Based on growth and colony morphology on MSA and Gram stain analysis, up to 60 isolates were obtained from each plaque specimen originating from every patient at all collection periods, with each isolate being confirmed as bacitracin-resistant, Gram-positive oral streptococci. Using primers specific for *S. mutans* or *S. sobrinus*, and testing genomic DNA from isolates obtained from the seven pediatric patients (Patients G, H, I, J, K, L and M), we identified 37 genotypic strains of *S. mutans*, two genotypic strains [K2 and K3 strains] of *S. sobrinus*, and seven non-MS strains during the entire study (pre-rehabilitation, 2–4 week post-rehabilitation, 6-month post-rehabilitation and 1-year post-rehabilitation) (18), including the appearance of six new MS strains found only at the 1-year collection (Fig. 1 and Table 2). For the five pediatric patients who completed the entire 1-year study, we identified 30 genotypic strains of *S. mutans*, two genotypic strains [K2 and K3 strains] of *S. sobrinus*, and five non-MS strains (Table 2). Several of the new strains identified at 1-year post-dental rehabilitation were found only as single isolates, or were highly-related to other genotypes that differ by only single bands in their AP-PCR genetic profiles (Fig. 1 and Table 2, e.g. compare L1c found only at the 1-year collection to dominant strain L1 and other minor MS strains L1a and L1b). Genomic DNA from several isolates were not amplified, or were weakly amplified using *S. mutans*-specific primers, and comprised seven additional genetic strains of bacitracin-resistant Gram-positive oral streptococci. As described in our previous report (18), representative isolates from these seven genotypic strains were subjected to 16S rRNA gene sequencing, and were identified as three strains of *S. gordonii*, three strains of *Streptococcus anginosus*, and one strain of *Granulicatella adiacens*, previously known as *S. adjacens* (27).

**Table 2:** Identification and percentage of mutans streptococci (MS) and oral streptococci genotypes at each visit

	Percentage of genotypes															
			
Patient visit	*S. mutans* 1										Other^2^				Number of genotypes	Dominant strain
Patient G	G1	G2	G4	G4a							G3					
Pre-treatment	18	56	16	3							6				5	G2 (56%)
Post-treatment (4 weeks)	0	98	0	0							2				2	G2 (98%)
Post-treatment (6 months)	0	100	0	0							0				1	G2 (100%)
Post-treatment (1 year)	0	100	0	0							0				1	G2 (100%)
																
Patient J	J1	J2	J2a	J2b	J2c	J3	J3a	J3b	J4	J5						
Pre-treatment	13	56	7	2	0	16	4	0	2	0					7	J2 (56%)
Post-treatment (2 weeks)	0	0	0	0	0	98	0	0	0	2					2	J3 (98%)
Post-treatment (6 months)	0	0	0	0	0	100	0	0	0	0					1	J3 (100%)
Post-treatment (1 year)	0	60	0	0	4	32	0	4	0	0					4	J2 (60%)
																
Patient K	K1^3^	K1a	K4								K2	K3				
Pre-treatment	90	7	0								3	0			3	K1 (90%)
Post-treatment (2 weeks)	57	0	0								0	43			2	K1 (57%), K3 (43%)
Post-treatment (6 months)	100	0	0								0	0			1	K1 (100%)
Post-treatment (1 year)	98	0	2								0	0			2	K1 (98%)
																
Patient L	L1	L1a	L1b	L1c	L2	L3										
Pre-treatment	96	0	0	0	2	2									3	L1 (96%)
Post-treatment (4 weeks)	96	2	2	0	0	0									3	L1 (96%)
Post-treatment (6 months)	100	0	0	0	0	0									1	L1 (100%)
Post-treatment (1 year)	89	0	6	3	2	0									4	L1 (89%)
															
Patient M	M2	M3	M3a	M5	M7	M8	M9				M1	M1a	M4^4^	M6^4^		
Pre-treatment	14	44	0	2	0	0	0				10	2	20	8	7	M3 (44%)
Post-treatment (4 weeks)	2	88	4	0	0	0	0				0	0	4	2	5	M3 (88%)
Post-treatment (6 months)	0	40	0	0	12	0	0				48	0	0	0	3	M3 (40%), M1 (48%)
Post-treatment (1 year)	0	93	0	0	0	2	2				3	0	0	0	4	M3 (93%)

1 Genotypes confirmed as *S. mutans* by conventional PCR with *S. mutans-*specific primers. Note that genotypes containing an ‘a’, ‘b’, or ‘c’ suffix as in G4a or J2a and J2b differ from the its matched comparison strains (in this case: G4 and J2) with the addition of one or more AP-PCR fragments (‘a’ suffix implies one additional band, ‘b’ suffix implies two additional bands and ‘c’ suffix implies three additional bands, all when compared to the AP-PCR profile of its matched genotypic strain). Note that individual MS genotypes were determined for comparison within each patient alone, and thus, comparisons of MS genotypes were conducted at only the intra-patient level.

2 Other genotypes are classified as non-MS oral streptococci, except genotypes K2 and K3 that were confirmed as *S. sobrinus* by conventional PCR with *S. sobrinus*-specific primers. Note that individual genotypes were determined for each patient alone, and thus, comparisons of MS genotypes were conducted at only the intra-patient level. Using 16S ribosomal RNA gene sequencing, we have made the following bacterial species identifications for genotypes classified in the non-MS oral streptococci group: G3=*S. gordonii*, M1=*S. anginosus*, M1b=*G. adiacens*, M4 and M6=both *S. gordonii*. To further characterize selected isolates that were difficult to identify by conventional PCR, acidification reactions (D-ribose, L-arabinose, D-mannitol, D-sorbitol, D-lactose, D-trehalose, inulin, D-raffinose, amidon (or starch), and glycogen; derived from API-20 Strep kit, Biomurieux SA) were also conducted and were compared against *S. mutans* strains ATCC 25175 and ATCC 35668, and *S. salivarius*.

3 Genotype K1 isolates obtained at the 2 week post-treatment collection did not yield robust PCR products using *S. mutans*-specific primers and conventional PCR; however, these isolates were counted in the MS group because they retained identical AP-PCR fingerprints when compared to other K1 isolates.

4 M4 and M6 isolates obtained at the pre-treatment collection did not yield robust PCR products using *S. mutans*-specific primers and conventional PCR. And small numbers of isolates from genotypes M4 (2 isolates) and M6 (1 isolate) obtained at the 2-week post-treatment collection yielded robust PCR products using *S. mutans*-specific primers and conventional PCR. We counted these three isolates from M4 and M6 obtained from the 2-week post-treatment collection as members of the non-MS group, because they retained metabolic fermentation profiles inconsistent with those obtained for several *S. mutans* strains.

Note: This table was shown in part in Palmer et al. (18) and is reproduced here with the kind permission of the American Academy of Pediatric Dentistry.

### Genotypic strains remain dominant and new minor strains appear at 1-year post-rehabilitation

As defined for the seven patients described in our previous report (18), the number of genotypic strains identified from any one patient over the entire collection period ranged from 3 to 9, or from any one visit from 3 to 7 (Fig. 1 and Table 2). In the five patients who completed all recall visits, including the 1-year recall, the highest numbers of strains were observed at the pre-rehabilitation collection, and dominant strains representing >44% of the population examined were prevalent at all post-rehabilitation collections. Four out of five patients (Patients G, K, L and M), who were all present at the 6-month recall visit, had only one dominant genotypic strain at 6 months post-rehabilitation, with the same genetic strain remaining at 1-year post-rehabilitation (Fig. 1 and Table 2). Interestingly, in four patients (Patients J, K, L and M), where one or two dominant strains were present at 6 months post-rehabilitation, the number of MS strains became more diverse at 1-year post-rehabilitation, with the emergence of new minor strains. Also, in almost all cases and collection times (with the exception of Patients M and J), there was only one dominant strain, each representing >56% of the strain population examined (Fig. 1 and Table 2). Genotype M1, which was dominant at 6 months post-rehabilitation, became greatly diminished at 1-year post-rehabilitation. In addition, in Patient J, genotype J2 was the dominant MS strain at pre-dental rehabilitation, disappearing beyond detection during the 2-week and 6-month collections, and then reappearing as the dominant strain at 1-year post-rehabilitation. Genotype J3 remained throughout the entire sampling period, and was the dominant MS strain during the 2-week and 6-month post-rehabilitation sampling times. The genotypic distribution patterns for the patient cohort were distinctive and unique for each patient; all comparisons of genotypes were conducted with strains collected within each individual patient, and not between patients. Inter-patient comparisons of MS strains were not conducted as part of this study.

### Xylitol-resistance determinations for dominant and minor MS genetic strains

**Fig. 2:**
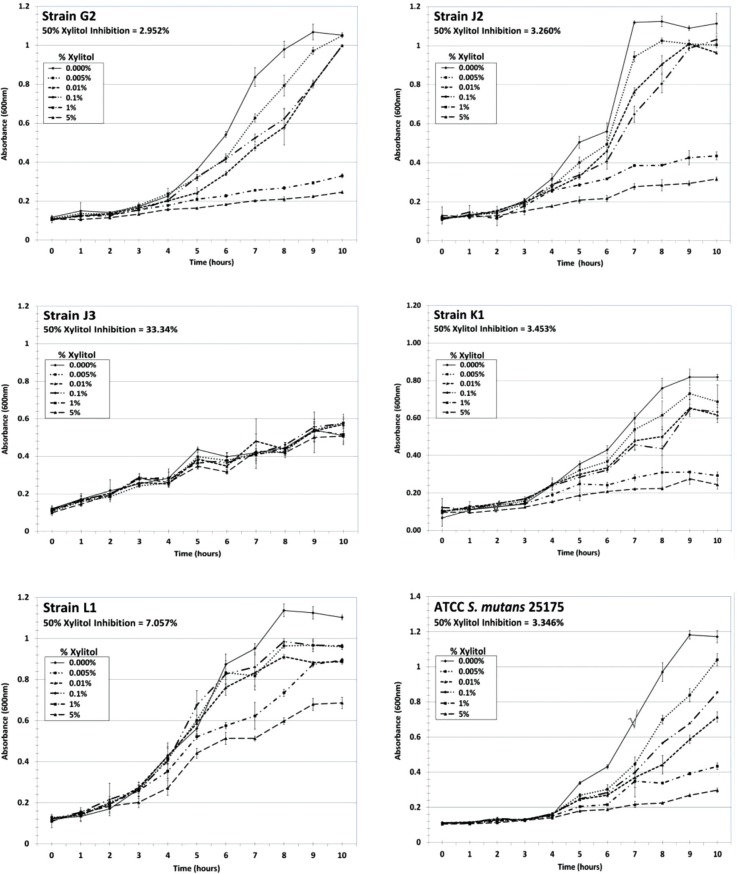
Xylitol inhibition curves for dominant MS strains and *S. mutans* ATCC 25175. Dominant MS strains G2, J2, J3, K1 and L1, and *S. mutans* ATCC 25175 were propagated in BHI for 24 hours at 37**°**C, and subsequently diluted in fresh BHI to an absorbance (600 nm) level of 0.1 to initiate logarithmic growth in the presence or absence of xylitol (final concentrations of 0%, 0.001%, 0.01%, 0.1%, 1% and 5% xylitol). Cultures were measured spectrophotometrically every hour for 10 hours and then at 24 hours, using four replicates per time point for each xylitol concentration. Plots were constructed and then curve fitted using cubic or quadratic models (23–25) to determine the theoretical xylitol concentrations for 50% inhibition of growth, using the peak absorbance of the 0% xylitol control as the normalization factor at 100%.

**Fig. 3:**
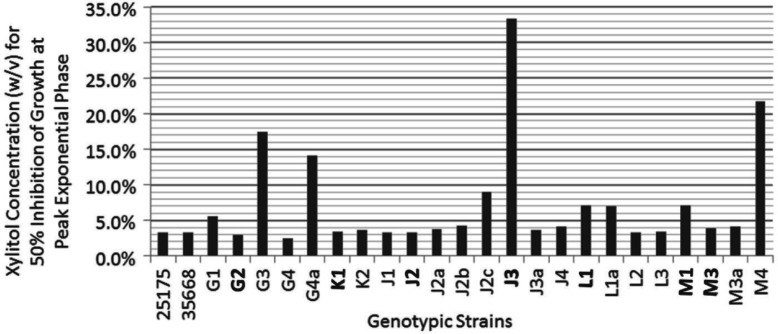
Bar graphs illustrating xylitol concentrations (w/v) for 50% inhibition of growth for dominant MS strains and select minor MS strains from Patients G, J, K, L and M. 50% inhibition values are also displayed for *S. mutans* ATCC 25175 and 35668. Dominant strains are in bold. Xylitol concentrations (w/v) for 50% inhibition of growth for: (1) *S. mutans* ATCC 25175 and 35668 are 3.35% and 3.30%, respectively; (2) Strains G1, G2, G3, G4, and G4a are 5.58%, 2.95%, 17.4%, 2.48% and 14.1%, respectively; (3) Strains K1 and K2 are 3.45% and 3.61%, respectively; (4) Strains J1, J2, J2a, J2b, J2c, J3, J3a, and J4 are 3.35%, 3.26%, 3.77%, 4.28%, 9.05%, 33.3%, 3.65% and 4.12%, respectively; (5) Strains L1, L1a, L2 and L3 are 7.06%, 7.00%, 3.35% and 3.43%, respectively; and (6) Strains M1, M3, M3a and M4 are 7.07%, 3.86%, 4.10% and 21.7%, respectively. M3 was the dominant strain in Patient M throughout the entire 1-year collection period. M1 was an additional co-dominant strain in Patient M at 6 months post-rehabilitation therapy.

Using an *in vitro* growth assay, we determined the 50% xylitol inhibition values for select MS strains to range between 2.48% and 33.3% xylitol, using the absorbance value of the 0% xylitol control at peak logarithmic phase (typically at 9–10 hours) as the 100% normalization value for each strain (Figs. 2 and 3). *S. mutans* ATCC strains 25175 and 35668, generally considered to be laboratory attenuated strains, retained 50% xylitol inhibition values of 3.35% and 3.30%, respectively (Figs. 2 and 3). The majority of the MS genetic strains analyzed (15 out of 23 strains from Patients G, J, K, L and M) exhibited 50% xylitol inhibition values ranging from 2.48% to 5.58%, similar to the 50% xylitol inhibition values shown by the *S. mutans* ATCC control strains. In patients where 1-year post-dental rehabilitation specimens were collected, dominant strains G2, K1, L1, and M3 exhibited 50% xylitol inhibition values of 2.95%, 3.45%, 7.06% and 3.86%, respectively (Figs. 2 and 3). In Patient J, where strain J3 was dominant at 6 months post-rehabilitation therapy, and strain J2 was dominant at 1-year post-therapy, strains J3 and J2 exhibited 50% xylitol inhibition values of 33.3% and 3.26%, respectively (Fig. 2). In the five pediatric patients who completed all post-rehabilitation recall visits [Patients G, J, K, L and M], the dominant MS genotypic strain at the 1-year post-rehabilitation collection exhibited 50% xylitol inhibition values similar or close to the values retained by the *S. mutans* ATCC control strains.

## Discussion

*S. mutans* is highly cariogenic because of its acidogenic and acid-tolerant properties, and its ability to generate biofilms and synthesize insoluble extracellular glycans. Distinct strains of *S. mutans* produce differential levels of glucosyltransferase enzymes (28), and caries-active individuals have been identified to contain greater numbers of MS genotypes with increased capacity to synthesize water-insoluble glycans (29). Thus, the genetic diversity of *S. mutans* and its corresponding differences in cariogenic potential has now been provided consideration as an important virulence factor of dental caries.

The original pilot study was undertaken to begin understanding the genetic diversity of *S. mutans* in children with S-ECC, and to define changes in the genotypic population within each of these patients following full-mouth dental rehabilitation therapy. In this current manuscript, using the remaining members of the original patient cohort, we now report the continued presence of dominant MS genotypic strains and the emergence of additional minor MS strains at 1-year post-dental rehabilitation. We also now describe the *in vitro* xylitol resistance of dominant and select minor MS genotypic strains.

### Genetic analysis and identification of MS strains

Studies by several investigative groups, including Napimoga et al. (30), Lembo et al. (31), and Baca et al. (32) have validated the use of AP-PCR in discriminating MS genotypes within individuals, and have formed the basis of our original pilot study. We also now find that AP-PCR profiles of MS isolates obtained at 1-year post-rehabilitation therapy can be reproducibly and reliably compared to profiles of MS isolates obtained earlier in the original study.

### MS colonization and genotypic diversity in children with severe early childhood caries

Mitchell et al. (33) suggested that the composition of strains within the MS population, or the acquisition and loss of specific MS strains, is dynamic in patients with S-ECC. We also observed similar MS population shifts in patients described in our study, for all time periods including the 1-year post-rehabilitation. As described in our previous report (18), Patient M may have undergone potential re-infection with genotypic strains from external sources at some point following the pre-rehabilitation collection, but prior to the 6-month collection date, as observed with the re-appearance of genotype M1 as a dominant strain at 1-year post-rehabilitation (Fig. 1 and Table 2). In addition, the re-appearance of genotype J2 at 1-year post-rehabilitation in Patient J, as well as the appearance of new minor MS strains in four patients (Patients J, K, L and M) at 1-year post-rehabilitation, may also be due to re-infection from external sources. Genotype J2, undetected at the 2–4 week and the 6-month collections, and genotype M1, undetected at the 2–4 week collection, may have been present at very low numbers below the threshold of detection. In the case of Patients J and M and other individuals with S-ECC, it is plausible that MS genotypes could be acquired vertically from their mother or horizontally between family members or extended care group. The diagnoses of ECC and S-ECC are associated with several risk factors including caregivers with high levels of MS and untreated carious lesions, frequent ingestion of sucrose-rich diets, and poor oral hygiene practices. In combination, these factors can result in MS colonization at earlier ages, with higher bacterial levels and greater number of MS genotypes than in caries-free children (29–34).

In this current study and as described in part in our previous report (18), our enrolled children with S-ECC, age 3–5 years, exhibited 3–7 genotypes prior to dental rehabilitation therapy, and most exhibited only 1–3 genotypes at 6 months post-therapy. Single genotypes, or the most dominant strains, have been identified in four out of five pediatric patients at 6-months post-rehabilitation therapy. Interestingly, for the remaining members of this patient cohort, the diversity of MS genotypes appeared to increase in four out of five patients [e.g. Patients J, K, L and M] at the 1-year post-therapy collection, with the appearance of six new minor MS strains.

As reported in part in our previous report (18), the results of our pilot study, including the 1-year post-dental rehabilitation data, are most similar to those of Pieralisi et al. (35) where we observe genetic diversity of MS strains in children with S-ECC, but also demonstrate that dental rehabilitation therapy results in the appearance of single dominant MS strains. Our primary objective was to define changes that occur in the composition of MS genetic strains in caries-active children, both prior to full-mouth dental rehabilitation therapy and at 1-year post-therapy. We were unable to determine if the diminution of the MS strains and the appearance of single dominant strains were due to the selective effects of the antimicrobial rinse, application of fluoride varnish, or the restorative procedure itself. Furthermore, studies using caries-free children were not conducted, but we acknowledge that this would represent an excellent experimental group in examining the selective effects of the antimicrobial rinse and fluoride treatment alone in the appearance of the dominant MS strains.

### Full-mouth dental rehabilitation therapy and effects on diversity of MS strains

In the original pilot study, we examined the genotypic strains of MS and other non-MS strains from seven pediatric patients that exhibited S-ECC, with an additional objective in evaluating the efficacy of the current regimen for full-mouth dental rehabilitation therapy. In most patients, dental rehabilitation therapy reduced the diversity of oral streptococci from several genotypic strains to 1–2 dominant strains by 6 months post-therapy. We presume that the treatment of the carious lesions, as well as the antimicrobial rinse and fluoride treatment, reduced the total bacterial numbers in all strains immediately following dental rehabilitation, but the effectiveness of the therapy was time-limited, with dominant strains appearing at 6 months post-rehabilitation. In most of the patients examined, and as described in our previous report (18), dental rehabilitation therapy eliminated several non-MS strains and allowed specific strains of the highly acidogenic *S. mutans* group to become the dominant strain. New minor MS strains appeared at 1-year post-therapy, potentially as a result of re-infection from the primary care giver and/or other external sources.

As with all of our pediatric dentistry patients, our participant cohort was provided with instructions for personal oral health care, including the use of brushing (twice daily with fluoridated toothpaste and rinse to control dental plaque). Since patient compliance with daily plaque control is unverifiable, we cannot rule out the possibility that the at-home use of toothpaste or rinses may have impacted the appearance of the MS dominant strains or the new minor strains at 1-year post-rehabilitation therapy.

We understand that we have limited numbers of patients in the pilot study and in the 1-year follow-up study, but believe that this work is statistically substantiated by Cheon et al. (36), who have used probabilities to determine the minimum number of MS isolates from an individual required to fully evaluate diversity of genotypes. Cheon et al. (36) determined theoretically that screening seven MS isolates from any one specimen collection was sufficient for the detection of up to four MS genotypes with a success probability of 78%. Our xylitol inhibition experiments indicate that most of the dominant MS strains are similar in xylitol resistance to the attenuated *S. mutans* ATCC control strains, with some strains being variably inhibited by xylitol *in vitro*. Moraes et al. (17) indicate that xylitol (40% solution) may not significantly suppress *S. mutans* counts or plaque accumulation in young children, as opposed to adults, potentially implicating the existence of xylitol-resistant MS strains. Our studies support Moraes et al. (17) and affirm the existence of variably inhibited MS strains, including more xylitol-resistant strains, and that these strains may have diverse cariogenic potential.
